# Lactobacillus gasseri liver abscess and bacteremia: a case report

**DOI:** 10.1186/s12879-021-06181-w

**Published:** 2021-06-02

**Authors:** David Ramos-Coria, Jorge Canto-Losa, Daniel Carrillo-Vázquez, Leonardo Carbajal-Morelos, Rodrigo Estrada-León, Eduardo Corona-Rodarte

**Affiliations:** Department of Internal Medicine, Instituto Nacional de Ciencias Médicas y Nutrición Salvador Zubirán, Vasco de Quiroga 15, Tlalpan, 14080 Mexico City, Mexico

**Keywords:** Bacteremia, Abscess, *Lactobacillus*, Pancreaticojejunostomy, Diabetes

## Abstract

**Background:**

Lactobacillus is a genus of Gram-positive non-spore-forming rods usually found in the microbiota of the oral cavity, gastrointestinal tract, and female genitourinary tract. Also, they are commonly used in the food industry as supplements and probiotics. Lactobacilli are normally considered non-pathogenic to the human body, however, under certain circumstances such as immunosuppression, they can cause severe infections, with only a few cases of bacteremia, infective endocarditis, pneumonia, meningitis, and intra-abdominal infections reported. Among these presentations, a pyogenic liver abscess is rather rare.

**Case presentation:**

We describe the case of a 59-year-old man with a history of diabetes mellitus and multiple abdominal surgeries with the latest being in 2014 presenting with bacteremia and multiple large pyogenic liver abscesses due to *Lactobacillus gasseri*, which did not appear to be related to the use of probiotics or immunosuppression.

**Conclusions:**

Given the high prevalence of diabetes mellitus and the increased use of probiotics, it is expected that in the future we will see an increase in infections caused by Lactobacilli. Medical management with antibiotics and percutaneous drainage were successful strategies for the treatment of this unusual case of pyogenic liver abscesses and bacteremia caused by *Lactobacillus gasseri*.

## Background

Lactobacilli are considered as non-pathogenic organisms of the human microbiota. These are Gram-positive rods, facultative anaerobes, non-spore-forming, and lactic acid-producing microorganisms frequently used in the food industry for the production of supplements and probiotics [1]. Under certain circumstances and with the presence of certain risk factors, they can be the cause of severe infections including bacteremia and infective endocarditis (IE) [2], liver abscesses, peritonitis, pulmonary infections, pyelonephritis, and meningitis, among others [3, 4]. Some of the main risk factors that have been described in *Lactobacillus* infections are diabetes mellitus (DM), structural heart disease [5], cancer (such as leukemia) [6], use of parenteral nutrition, use of broad-spectrum antibiotics, chronic kidney disease (CKD), neutropenia, solid-organ transplantation [7], chemotherapy, HIV and steroid use [3]. Predisposing factors like dental manipulations, poor dental hygiene, intravenous drug use, history of abdominal surgeries, and use of probiotics, are consistently reported [8].

The incidence of bacteremia and liver abscesses due to Lactobacilli is rare, with only 10 cases reported in the literature [3, 9–17]. To our knowledge, this is the first case of a patient with a liver abscess and bacteremia caused by *Lactobacillus gasseri.*

## Case presentation

A 59-year-old man with a history of open cholecystectomy (2013), acute necrotizing pancreatitis, complicated with pancreatic fistula, distal pancreatectomy, splenectomy, and pancreaticojejunal anastomosis (2014), causing type 3C DM (2016) and adhesive small bowel occlusion (2017) was admitted for weakness, fever, and abdominal pain. Upon arrival, he had tachycardia (heart rate of 102 bpm) and tachypnea (respiratory rate of 32 rpm). He referred pain at the right hypochondrium and having uninvestigated intermittent melena for the past 2 years. Alcohol, intravenous drug abuse, or herbal product use was denied. Physical examination revealed a well orientated, mildly dehydrated patient with generalized jaundice. Bowel sounds were decreased in frequency and intensity, and the abdomen was tympanic at percussion. Laboratory tests showed white blood cells 24.8 x 10^3^, neutrophils 89.7%), anemia (hemoglobin 8.8 g/dL), diabetic ketoacidosis, acute kidney injury (AKI), and hypertransaminasemia with a cholestatic pattern (total bilirubin, 1.51 mg/dL; direct bilirubin, 0.94 mg/dL, indirect bilirubin 0.57 mg/dl, aspartate aminotransferase, 52 IU/L; alanine aminotransferase, 127 IU/L; alkaline phosphatase, 435 IU/L); CRP 20.65 mg/dL; Hepatitis B surface antigen, hepatitis B e-antigen, and hepatitis C virus antibody and HIV screening tests were all negative.

**Fig. 1:**
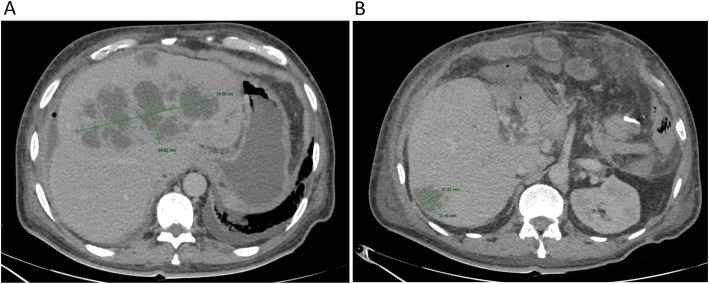
Computed Tomography (CT) scan showing an enlarged liver with (A) multiple heterogeneous hypodense fluid-filled lesions compatible with liver abscesses on both hepatic lobes. The largest abscess was localized on the liver segment VIII measured 14.5 cm × 5.4 cm. Another small abscess was present on liver segment IV. (B) A smaller abscess (3.1 cm × 2.7 cm) was present on the liver segment VI. Note the surgical absence of the gallbladder and the spleen

Acute cholangitis was suspected and computed tomography (CT) of the abdomen was performed (Fig. 1), showing a non-cirrhotic liver with a cephalocaudal length of 18 cm, with multiple heterogeneous lesions in both hepatic lobes, with attenuation in liquid ranges, compatible with liver abscesses, the largest measuring (14.5 cm × 6.5 cm) in the left lobe and segment VIII with bile duct dilation in segments II, III and VIII, ascites and chronic portal vein thrombosis with cavernomatous degeneration.

The patient developed progressive hypotension and hyperlactatemia requiring norepinephrine infusion (maximum dose of 0.19 mcg/kg/min). He was hospitalized and empiric broad-spectrum antibiotic therapy was immediately initiated with intravenous meropenem and vancomycin. Septic shock secondary to liver abscesses was diagnosed. The largest hepatic abscess, localized on liver segment VIII, was managed by CT-guided percutaneous drainage and 100 ml of purulent material was obtained and sent for culture. Blood cultures were also obtained at admission. After 48 h, both the liver abscess culture and blood cultures revealed lactic-acid forming bacteria with a subsequent characterization of *Lactobacillus gasseri* by MALDI-TOF mass spectrometry. Antibiogram unveiled susceptibility to Penicillin G, thus the antimicrobial regimen was modified to intravenous penicillin G, 24 million IU QD.

**Fig. 2:**
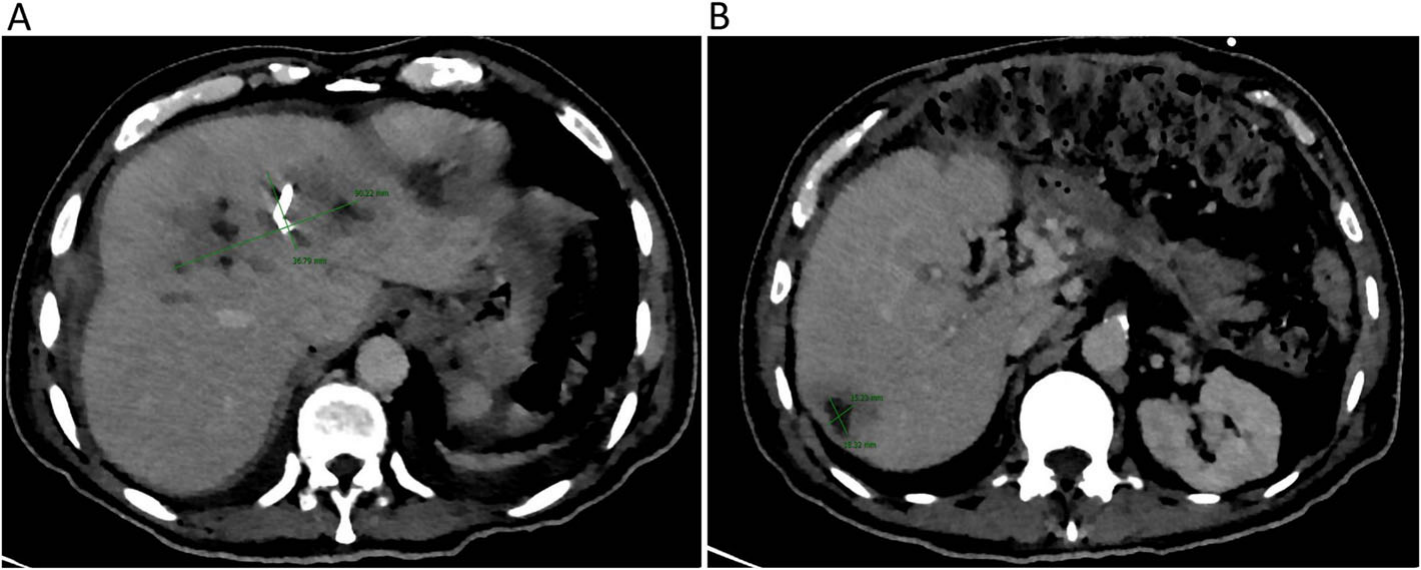
CT scan was taken 10 days after the CT-guided percutaneous drainage was performed. (A) The distal portion of a 10 Fr Dawson-Müller multipurpose drainage catheter can be seen in the interior of the abscess localized on liver segment VIII. Gas is present in the interior of the abscess because of the multipurpose drainage catheter insertion. Note the smaller size of the abscess (12.1 cm × 4.2 cm) compared with the first CT scan (14.5 cm × 5.4 cm). (B) The abscess on liver segment VI also decreased in size with 10 days of antibiotic therapy, measuring 1.8 cm × 1.5 cm (previously 3.1 cm × 2.7 cm)

**Fig. 3:**
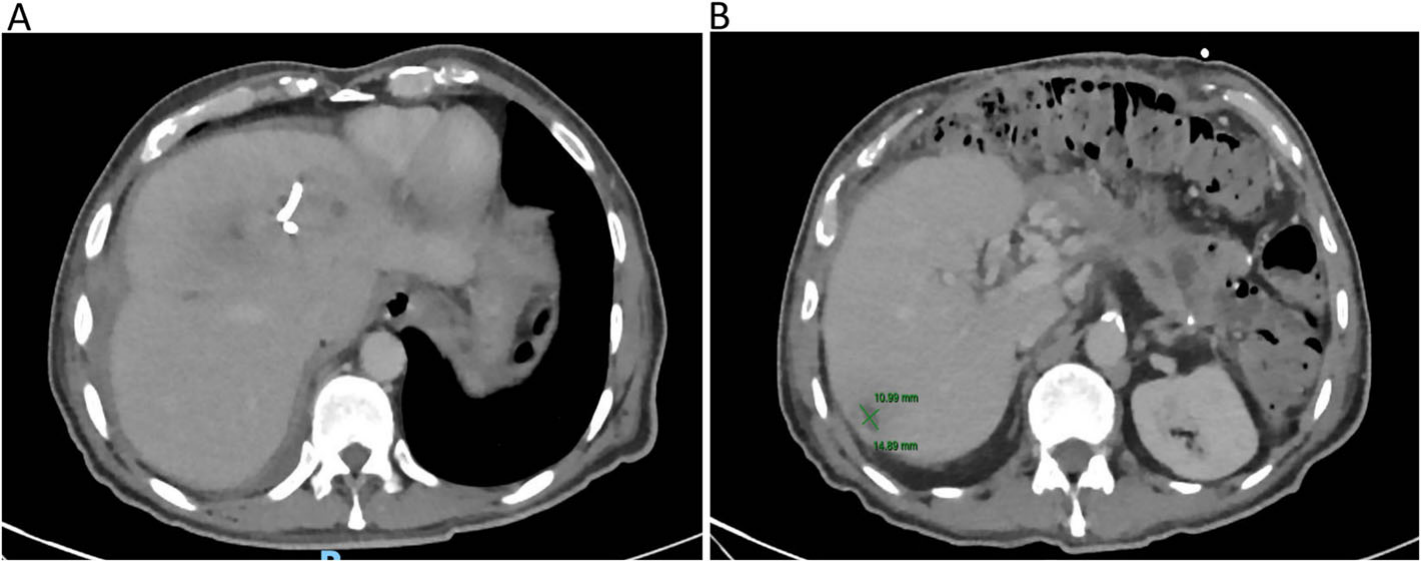
CT scan was taken 26 days after the CT-guided percutaneous drainage was performed. (A) Note the dramatic change in the size of the abscess localized on liver segment VIII. (B) The abscess localized on liver segment VI continued to decrease in size (1.4 cm × 1.0 cm) with antibiotic therapy

An esophagogastroduodenoscopy was performed revealing a Los Angeles grade “A” esophagitis and mild portal gastropathy, without esophageal varices. A double-dose proton pump inhibitor was continued (omeprazole 40 mg every 12 h). A transthoracic echocardiogram was performed without any findings of IE. A subsequent CT of the abdomen (Fig. 2) showed an adequate response to treatment with a significant decrease in the size of the three abscesses. The patient remained afebrile, without signs of gastrointestinal bleeding or evidence of a systemic inflammatory response. Four weeks after admission a third CT was performed (Fig. 3), showing an additional decrease in the size of the abscesses. Due to clinical stability, after source control and 24 days of effective intravenous treatment the patient was discharged. Oral amoxicillin was given for a 4-week period with total clinical recovery and imaging improvement.

## Discussion and conclusions

The incidence of liver abscess varies depending on the geographic area and the studied population. Globally, the incidence rate is 3.6 cases per 100,000 inhabitants. The main risk factors are advanced age (> 65 years) and male gender [18]. Its etiology has changed locally and globally, largely due to the high incidence and prevalence of DM in the Western world [19, 20]. Clinical presentation is variable and can range from fever (89%), right upper quadrant abdominal pain (72%), chills (69%), nausea (43%), vomiting (32%), weight loss (26%), jaundice (21%), headache (17%), myalgia (11%), and diarrhea (10%). The frequency of liver abscesses complicated with septic shock ranges from 5.6–16% [21]. The most frequently isolated microorganisms are *E. coli, Klebsiella pneumoniae, Proteus vulgaris, Enterobacter*, and multiple microaerophilic anaerobes (such as *Streptococcus anginosus*), therefore, empirical broad coverage of a probable polymicrobial infection is indicated. Other less frequent agents are *Salmonella, Haemophilus, Yersinia,* and *Listeria* [22].

Bacteria can invade the liver through ascending cholangitis, pylephlebitis (infection of the portal vein), the hepatic artery (secondary to bacteremia), contiguity of a nearby infectious process, or traumatic implants through the abdomen [23]. The first three mechanisms of invasion are compatible with the risk factors and characteristics of this patient. The mechanism by which the invasion of *Lactobacillus* is achieved remains unknown. A preponderant role in the pathophysiology of the disease is micro and macroangiopathy secondary to DM. Atherosclerotic lesions and advanced glycosylation products induce glycation of structural matrix proteins in cell basement membranes, increasing vascular permeability, responsible for endothelial injury and intestinal bacterial translocation [24]. During the diagnostic approach of this patient, exhaustive clinical reviews (dental, upper endoscopy, and echocardiography) were performed, without evidence of any predisposing lesion, except for the surgically modified biliodigestive anatomy.

Liver abscesses caused by *Lactobacillus* strains have been reported even in the absence of relevant probiotic consumption [17] or hepatopancreatobiliary instrumentation [9, 12, 14]. Table 1 shows *Lactobacillus* liver abscess cases reported in literature. It should be noted that in up to 40% of cases, it is not possible to demonstrate a cause and such cases remain cryptogenic [25]. Percutaneous or surgical drainage should be performed if an abscess is larger than 5 cm or in patients with persistent clinical symptoms and tenacious evidence of abscess on imaging. In eight of the ten reported cases, drainage, either percutaneous or surgical, was performed with excellent results and no fatal outcomes. There were no reported fatal outcomes in both extended series, but there was an increase in the hospital stay when compared to other causes of pyogenic liver abscess (mean of 48 days vs mean of 16 days) [3, 17].

**Table 1:** Summary table of published *Lactobacillus* liver abscess cases

Reference (year)	Age (years)/sex	Comorbidities	Risk factors	Species	Treatment
Isobe H. (1990) [9]	75/M	Hepatocellular carcinoma, Parkinson’s disease	Intratumoral ethanol injection therapy for HCC	*L. plantarum*	Antibiotics
Larvol L. (1996) [10]	39/M	DM, pancreatitis chronic pancreatitis, choledochoduodenostomy	N/A	*L. Acidophilus*	Antibiotics
Rautio M. (1999) [11]	74/F	DM, HTN	Heavy dairy consumption	*L. rhamnosus*	Percutaneous drainage plus antibiotics
Notario R. (2003) [12]	73/F	DM	N/A	*L. rhamnosus*	Surgical drainage plus antibiotics
Cukovic-Cavka S. (2006) [13]	27/M	Crohn’s disease	Steroid use	*L. acidophilus*	Percutaneous drainage plus antibiotics
Burns D. (2007) [14]	51/F	None	None	*L. paracasei*	Percutaneous drainage plus antibiotics
Chan JF. (2009) [15]	74/M	DM, HTN, remote history of tonsillar carcinoma	Mirizzi syndrome	*L. rhamnosus*	Percutaneous drainage, cholecystectomy plus antibiotics
Sherid M. (2016) [3]	82/F	DM, HTN, CKD, Cholecystectomy	Cholecystectomy, probiotic use	*Lactobacillus spp.*^a^	Percutaneous drainage plus antibiotics
Pararajasingam A. (2017) [16]	65/F	DM, HTN, hypercholesterolemia	Probiotic use	*L. paracasei*	Percutaneous drainage plus antibiotics
Abdillahi O. (2019) [17]	46/M	DM	None	*Lactobacillus spp.*^a^	Percutaneous drainage plus antibiotics
Ramos-Coria D. (2020) (current case)	59/M	DM, cholecystectomy, chronic pancreatitis, distal pancreatectomy plus pancreaticojejunostomy	Biliary tract instrumentation	*L. gasseri*	Percutaneous drainage plus antibiotics

*M* male, *F* female, *DM* Diabetes Mellitus, *HTN* Hypertension, *CKD* Chronic Kidney Disease, *HCC* Hepatocellular Carcinoma, *N/A* Not available

^a^The exact strain was not identified

Bacteremia and liver abscesses due to *Lactobacillus* are extremely rare. In a series from Finland, 89 cases of *Lactobacillus* bacteremia were identified. Of these, the species characterization of 47 were achieved; 25 being *L. rhamnosus* and 22 other species (*L. fermentum 20%, L. casei 15%, L. gasseri, L. zeae*). Immunosuppression, previous prolonged hospitalization, and previous surgical interventions were identified as independent risk factors [26]. *Lactobacillus* bacteremia implies a dark prognosis, with an associated mortality of 30% [25].

The clinical picture in the reported cases is similar to pyogenic liver abscesses caused by other microorganisms. Fever is nearly a rule, as well as abdominal pain or discomfort. Manifestations as tachycardia, fatigue, weakness, hyporexia, nausea, and vomiting are frequent, as well as local signs such as right upper quadrant tenderness and hepatomegaly. Less common manifestations for instance lower extremity atrophy, purpura fulminans, and right pleural effusion have been reported [10–14]. Regarding laboratory alterations, leukocytosis and neutrophilia have been present in all cases. Anemia is also habitual, reflecting the subacute nature of the condition, additionally to an elevation of acute phase reactants such as ESR, CRP, and ferritin. Thrombocytosis and alteration of liver chemistry with a predominantly cholestatic pattern may also be present.

The antibiotic susceptibility of Lactobacilli is variable. The most commonly used regimens are penicillins (penicillin and ampicillin) with or without aminoglycosides. In a retrospective study of 200 cases of *Lactobacillus spp* infections, the most commonly used regimens included penicillin monotherapy (*n* = 35), penicillin therapy combined with aminoglycoside (*n* = 20), and cephalosporins in monotherapy (*n* = 16). Clindamycin (90.0%) and erythromycin (94.3%) had the highest sensitivity, meanwhile, penicillin had 63.6%. Vancomycin had the highest resistance rates (sensitive only in 22.5% of cases) [27]. Due to the rarity of infections caused by Lactobacilli, clinical experience and clinical trials on the preferred antimicrobial treatment regimens are lacking.

Severe *Lactobacillus* infections are rare entities, and their uncertain significance implies a targeted search for risk factors capable of explaining the etiology, as in liver abscesses, where poorly controlled DM or hepatopancreatobiliary surgical manipulation are the main risk factors. Given the rising prevalence of population with risk factors for these kinds of infections, we should expect a rise in these cases; further study is needed to better understand risk factors, pathogenesis, and antibiotic susceptibility of *Lactobacillus spp.* bacteremia.

## Data Availability

Data sharing is not applicable to this article as no datasets were generated or analyzed during the current study.

## Abbreviations

CRP: C Reactive Protein
HIV: Human Immunodeficiency Virus
CT: Computed tomography
IU: International Units
HBV: Hepatitis B Virus
HCV: Hepatitis C Virus
M: Male
F: Female
DM: Diabetes Mellitus
HTN: Hypertension
AKI: Acute Kidney Injury
CKD: Chronic Kidney Disease
HCC: Hepatocellular Carcinoma
N/A: Not available
